# Extensive thrombosis revealing a Leinden factor mutation

**DOI:** 10.1016/j.radcr.2026.06.015

**Published:** 2026-06-30

**Authors:** Youssef Bouktib, Ayoub EL Hajjami, Badr Boutakioute, Meriem Ouali Idrissi, Najat Cherif Idrissi EL Guennouni

**Affiliations:** Department of radiology Arrazi, Mohammed VI University Hospital, Marrakesh, Morocco

**Keywords:** Radiology, CT imaging, MRI, Contrast-enhanced CT, Venous thrombosis imaging, Vascular imaging, Leinden factor mutation

## Abstract

Factor V Leiden (FVL) mutation is the most common hereditary thrombophilia and a well-established predisposing factor for venous thromboembolism, while its role in arterial thrombosis remains incompletely understood. We report the case of a 30-year-old postpartum woman who presented with extensive multifocal thromboembolic disease, including bilateral pulmonary embolism, renal infarction, left ventricular apical thrombus in the setting of peripartum cardiomyopathy (LVEF 20%), and acute ischemic stroke—all attributed to an underlying heterozygous Factor V Leiden mutation confirmed by PCR. Multimodality imaging, including CT pulmonary angiography, transthoracic echocardiography, and brain MRI, was central to establishing the full extent of thromboembolic disease and guiding anticoagulation therapy. This case highlights the pivotal role of imaging in diagnosing multifocal thromboembolism and underscores the importance of systematic thrombophilia workup in young patients presenting with unexplained or extensive thrombotic events, particularly in the postpartum period.

## Introduction

Resistance to activated protein C (APC) is a common cause of venous thromboembolism. In approximately 95% of cases, this resistance results from a point mutation in the factor V gene—a key procoagulant cofactor required for the conversion of prothrombin to thrombin—rendering it resistant to inactivation by APC. This genetic alteration produces an abnormal variant known as Factor V Leiden (FVL) [1].

The Factor V Leiden mutation and the consequent APC resistance are strongly associated with venous thrombotic events, particularly deep vein thrombosis and pulmonary embolism. However, the implication of this mutation in arterial thrombosis remains controversial and not yet fully elucidated.

We report the case of a young woman who presented with multiple thromboembolic manifestations, including pulmonary embolism, renal infarction, a left ventricular apical thrombus, and ischemic stroke, ultimately revealing an underlying Factor V Leiden mutation [1,2].

## Case report

A 30-year-old woman was admitted to the emergency department for acute hemoptysis and dyspnea that appeared during the fourth month postpartum. The patient was nonobese, with no previous cardiac history, and presented with signs of congestive heart failure on admission.

Her family history was negative for thrombotic disorders. Regarding her past medical history, she had no previous miscarriages, no episodes of deep vein thrombosis, and no history of prolonged bed rest or hospitalization.

On physical examination, she was tachycardic (119 beats/min) and polypneic (25 breaths/min). Cardiac auscultation revealed a diastolic murmur at both the mitral and aortic foci, with clinical signs of right heart failure. Peripheral vascular examination was unremarkable.

A chest X-ray demonstrated cardiomegaly (cardiothoracic index = 0.65) with blunting of the right costophrenic angle. The electrocardiogram showed sinus rhythm, left ventricular hypertrophy (LVH), and secondary repolarization abnormalities.

Given the high clinical suspicion of pulmonary embolism, a CT pulmonary angiography was performed. It confirmed a bilateral pulmonary embolism involving the apical branches, associated with bilateral pleural effusion (moderate on the right, minimal on the left), cardiomegaly, and an apical thrombus in the left ventricle. The abdominal cuts revealed foci of renal and hepatic infarction (Fig. 1, Fig. 2).

**Fig. 1:**
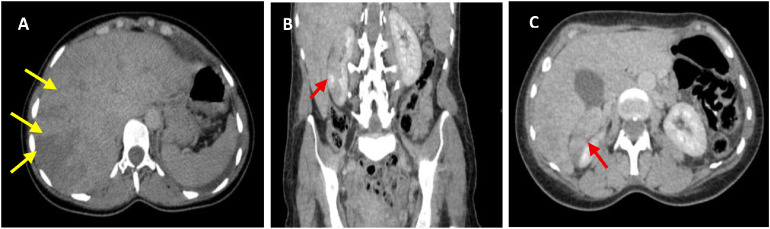
Abdominal CT scan in axial and coronal planes: (A) Hepatic perfusion abnormalities and (B and C) Foci of right renal infarction.

**Fig. 2:**
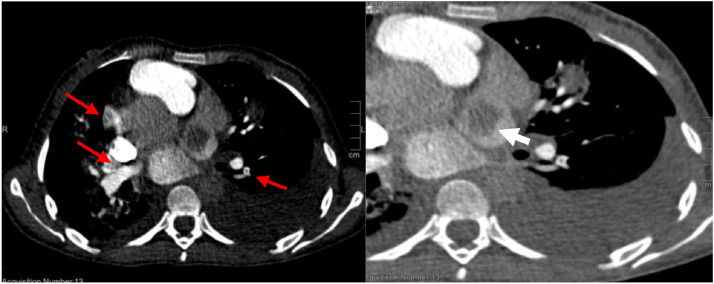
CT pulmonary angiography (angio-CT), axial sections. (A) Hypodense filling defects in the right and left main pulmonary arteries (red arrows), confirming bilateral proximal pulmonary embolism and (B) Axial section at the cardiac level showing a hypodense endoluminal thrombus within the left ventricular apex (white arrow).

Before initiating anticoagulation, the patient’s main laboratory parameters were as follows: erythrocytes 4.27 × 10⁶/µL, hemoglobin 11.5 g/dL, hematocrit 40%, platelets 259 × 10³/µL, D-dimers positive, and INR 1.19. Renal and hepatic function tests were within normal limits (Table 1).

**Table 1:** Laboratory findings and reference ranges.

Parameter	Patient value	Reference range
Erythrocytes	4.27 × 10⁶/µL	3.8-5.2 × 10⁶/µL
Hemoglobin	11.5 g/dL	12-16 g/dL
Hematocrit	40%	36%-46%
White blood cells	7800/mm³	4000-10,000 /mm³
Platelets	259 × 10³/µL	150-400 × 10³/µL
Prothrombin time (PT)	95%	70%-100%
INR	1.19	0.8-1.2
aPTT	32 s	28-35 s
Fibrinogen	3.4 g/L	2-4 g/L
Protein C activity	72%	70%-140%
Protein S activity	64%	60%-130%
Antithrombin III	95%	80%-120%
Factor V Leiden mutation	Positive (heterozygous)	Negative
D-dimers	Positive	< 500 ng/mL

Transthoracic echocardiography (TTE) revealed a dilated left ventricle with severely depressed systolic function (LVEF = 20%, Simpson’s method) due to global hypokinesia. A mobile, echogenic apical thrombus measuring 35 × 19 mm was visualized. The right chambers were also dilated, with severe right ventricular dysfunction (fractional area change = 29%, TAPSE = 14 mm, S′RV = 7 cm/s). Pulmonary artery systolic pressure was estimated at 30 mmHg. Valvular assessment revealed moderate aortic regurgitation and moderate mitral regurgitation (Fig. 3).

**Fig. 3:**
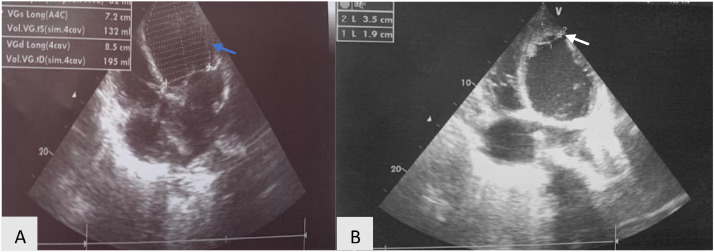
Transthoracic echocardiography (TTE). (A) Apical 4-chamber view demonstrating marked left ventricular (LV) dilatation with severely reduced systolic function (LVEF = 20% by Simpson's biplane method) and right ventricular enlargement (white arrows) and (B) Apical 2-chamber view showing a mobile, highly echogenic apical thrombus measuring 35 × 19 mm within the dyskinetic left ventricle (blue arrow).

In search of the thromboembolic source and to exclude occult malignancy, venous and abdominal ultrasound examinations were performed. Venous Doppler of the lower limbs ruled out deep vein thrombosis.

An ischemic or embolic etiology was investigated. The patient had no history of angina or myocardial infarction, and echocardiography showed no regional wall motion abnormalities suggestive of coronary artery disease. Therefore, the cardiomyopathy was considered nonischemic.

Given the occurrence of multiple thromboembolic events in a young woman without traditional cardiovascular risk factors, a thrombophilia screening was conducted to identify a potential underlying hypercoagulable state. The investigation included testing for protein C and S activity, antithrombin III levels, prothrombin gene mutation (G20210A), and Factor V Leiden mutation. The results confirmed the presence of a heterozygous Factor V Leiden mutation, responsible for resistance to activated protein C (APC), thereby predisposing the patient to recurrent venous and systemic thromboembolic events.

During hospitalization, the patient developed a transient neurological deficit with dysarthria and central facial palsy. Brain MRI angiography demonstrated an ischemic lesion (Fig. 4).

**Fig. 4:**
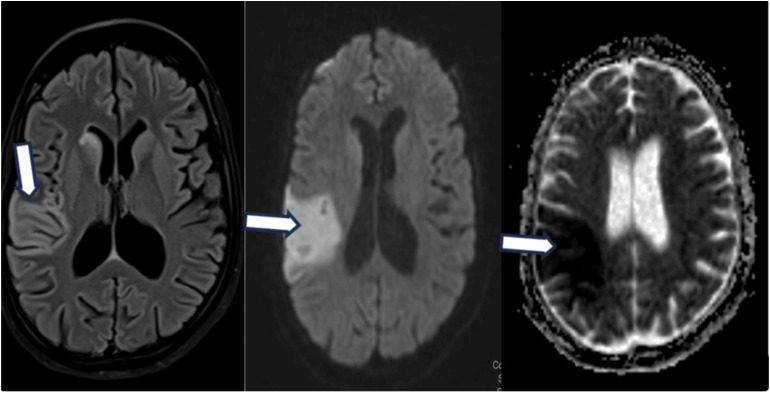
Brain MRI in FLAIR (A), diffusion-weighted (B), and ADC (C) sequences showing an established ischemic stroke in the deep and superficial sylvian territory, appearing hyperintense on FLAIR with restricted diffusion (white arrow).

Therapeutic anticoagulation was started with enoxaparin and continued later with rivaroxaban. Since our patient was hemodynamically stable, there was no indication for systemic thrombolysis.

## Discussion

Among congenital disorders of hemostasis, familial thrombophilia due to a Factor V mutation, known as Factor V Leiden (named after the Dutch city where the anomaly was first identified), holds particular clinical importance because of its high prevalence in the general population and its potential to cause severe thrombotic complications [1,2].

This genetic anomaly was first described in 1993 by Dahlbäck et al. [2] and subsequently characterized at the molecular level in 1994 by Bertina et al. [3]. The mutation involves exon 10 of the Factor V gene, resulting in the substitution of arginine by glutamine at position 506 (R506Q). This substitution eliminates one of the cleavage sites for activated protein C (APC) on Factor V and Factor Va, leading to resistance to APC-mediated inactivation and consequently to a loss of its anticoagulant cofactor function.

As a result, Factor V Leiden can no longer act as a cofactor for APC (in the presence of phospholipids, calcium, and protein S), leading to persistence of activated Factor V in the circulation and a state of hypercoagulability. Since protein C is a key physiological anticoagulant through its inhibitory action on Factors V and VIII, this defect contributes to a pro-thrombotic tendency [4].

The prevalence of Factor V Leiden varies according to ethnicity and geography: 3%-5% in Caucasian populations [5], approximately 6% in the United States [6], and up to 10% in eastern France, while it is virtually absent in Asian populations. The mutation accounts for 15%-20% of venous thromboses [7]. Arterial thrombosis, however, remains rare, with few cases reported before 1999.

The disorder is transmitted as an autosomal dominant trait, most often revealed by superficial or deep venous thrombosis, and more rarely by pulmonary embolism or by unusual localizations such as cerebral, mesenteric, or portal venous thrombosis. Thrombotic events are usually spontaneous and recurrent, occurring in individuals under 45 years of age, mostly in the heterozygous state; the thrombogenic risk is approximately ten-fold higher in homozygous carriers [1].

Predisposing factors are often absent, which justifies a systematic etiological investigation to detect congenital abnormalities-antithrombin III, protein C or S deficiency, Factor V mutation, prothrombin G20210A mutation, or hyperhomocysteinemia-as well as acquired causes such as lupus anticoagulant or antiphospholipid antibodies, both conferring high thrombotic risk [8,9].

Molecular testing (PCR amplification of genomic DNA) enables definitive diagnosis. While venous thrombosis can be explained by venous stasis and hypercoagulability, arterial thrombosis generally requires an endothelial injury capable of triggering platelet activation and thrombus formation [10].

Until the late 1990s, few arterial events related to Factor V Leiden were described, and several large studies failed to demonstrate a significant increase in the prevalence of the mutation among patients with myocardial infarction or ischemic stroke [11,12]. However, isolated reports have suggested a possible association with early-onset arterial thrombosis, particularly in the cerebral and coronary circulations [6].

Unusual localizations have also been described, including mesenteric and small-bowel infarction [13,14]. Arterial thrombosis without atherosclerotic stenosis may also occur in association with deficiencies in protein C, antithrombin III, or protein S, or with prothrombin gene mutations, especially in young women. Frequently, multiple thrombophilic factors coexist, amplifying clinical expression [15,16].

The mutation may remain clinically silent, but the risk of developing thrombosis is seven-fold higher in heterozygotes and eighteen-fold higher in homozygotes [17]. This risk increases with age, pregnancy, and oral contraceptive use. The mutation may coexist with other prothrombotic abnormalities such as protein C/S or antithrombin deficiency, hyperhomocysteinemia, antiphospholipid antibodies, or even a positive rheumatoid factor [18,19].

PCR-based detection of the mutation (confirmatory diagnosis) may follow a phenotypic screening test-APC resistance assay-performed on diluted plasma deficient in Factor V [19]. Testing is indicated in the following scenarios:

• A first spontaneous proximal DVT and/or PE before age 60.
• A first thromboembolic episode in a woman of childbearing age (spontaneous or provoked).
• Recurrent proximal DVT and/or PE.
• Recurrent unprovoked distal DVT before age 60.

In familial contexts, first-degree relatives may be screened in cases of homozygous FVL or combined heterozygous FVL-FII mutation. For heterozygous carriers, family testing is mainly considered in women of reproductive age, after thorough counseling on the implications for contraception and pregnancy.

Recent data show that, apart from a slightly higher incidence of VTE and shorter VTE-free survival, penetrance and severity do not differ significantly between heterozygous and homozygous carriers, suggesting that management and prevention strategies should not vary according to genotype [18].

Approximately 90% of APC resistance cases are due to the Factor V Leiden mutation, confirmed by molecular testing. The remaining acquired forms may occur during pregnancy, oral contraceptive use, or antiphospholipid syndrome, or may correspond to other Factor V variants (eg, Cambridge, Hong Kong). A positive APC-resistance test in the absence of FVL is still considered a marker of hypercoagulability [18].

Factor V Leiden is the most common inherited cause of venous thromboembolism, accounting for 10%-20 % of cases. Most carriers are heterozygous, with a 3- to 5-fold increased risk, whereas homozygotes have a fifty-fold risk. Clinical manifestations such as DVT or PE typically occur spontaneously or following triggering events (bed rest, surgery, estrogen therapy, pregnancy), in young adults or even adolescents.

Combined thrombophilic defects, particularly co-inheritance with the prothrombin G20210A mutation, further increase thrombotic risk. Therefore, once the diagnosis of Factor V Leiden is established, family screening is recommended to identify other carriers and to search for associated abnormalities. Preventive measures rely on patient education, risk-factor avoidance, and careful management of pro-thrombotic situations (contraception, pregnancy, hormone therapy). The recurrence of thromboembolic events warrants a case-by-case discussion of long-term anticoagulation [19].

The management of LV thrombosis in these patients remains challenging. Warfarin therapy is recommended by both the ESC and AHA guidelines as the treatment of choice following myocardial infarction [20]. However, recent reports have described successful resolution of LV thrombi with direct oral anticoagulants (DOACs) such as dabigatran or rivaroxaban [20]. Further studies are required to confirm their efficacy and safety in this indication.

In some patients, heparin resistance complicates management-defined as failure to achieve an APTT > 1.5 × baseline despite > 35,000 U/d of unfractionated heparin. When this occurs, argatroban may be useful, although rare cases of argatroban resistance have been reported [20], Rivaroxaban, approved for DVT treatment and secondary VTE prevention [20], may represent an effective alternative for patients with LV and venous thrombosis associated with protein C deficiency or Factor V Leiden.

The decision to screen for the FVL mutation before prescribing estrogen-progestin contraception or during pregnancy in women with a positive family history remains individualized and should be guided by a careful risk-benefit assessment.

## Conclusion

This case illustrates an unusual and multifocal thrombotic presentation that revealed an underlying Factor V Leiden mutation. The simultaneous involvement of pulmonary, cardiac, abdominal, and cerebral territories emphasizes the need to consider inherited thrombophilia when thrombotic events are extensive or lack a clear provoking factor. Early identification of such atypical patterns is essential to ensure accurate diagnosis and appropriate therapeutic planning.

Targeted anticoagulation with a direct oral agent contributed to the stabilization of this patient’s condition and helped prevent further embolic complications. Ongoing clinical follow-up and careful management of additional modifiable risk factors remain important to reduce the likelihood of recurrence.

## Ethical statement

This publication complies with institutional ethical standards, and no additional approval from an ethics committee was required for this single-patient case report.

## Patient consent

Written informed consent for publication of this case report and associated images was obtained from the patient.
